# Late Endosomal Cholesterol Accumulation Leads to Impaired Intra-Endosomal Trafficking

**DOI:** 10.1371/journal.pone.0000851

**Published:** 2007-09-05

**Authors:** Komla Sobo, Isabelle Le Blanc, Pierre-Philippe Luyet, Marc Fivaz, Charles Ferguson, Robert G. Parton, Jean Gruenberg, F. Gisou van der Goot

**Affiliations:** 1 Department of Microbiology and Molecular Medicine, University of Geneva, Geneva, Switzerland; 2 Department of Biochemistry, University of Geneva, Geneva, Switzerland; 3 Institute for Molecular Bioscience, Centre for Microscopy and Microanalysis, School of Biomedical Sciences, The University of Queensland, Brisbane, Australia; 4 Ecole Polytechnique Fédérale de Lausanne, Lausanne, Switzerland; Duke University Medical Center, United States of America

## Abstract

**Background:**

Pathological accumulation of cholesterol in late endosomes is observed in lysosomal storage diseases such as Niemann-Pick type C. We here analyzed the effects of cholesterol accumulation in NPC cells, or as phenocopied by the drug U18666A, on late endosomes membrane organization and dynamics.

**Methodology/Principal Findings:**

Cholesterol accumulation did not lead to an increase in the raft to non-raft membrane ratio as anticipated. Strikingly, we observed a 2–3 fold increase in the size of the compartment. Most importantly, properties and dynamics of late endosomal intralumenal vesicles were altered as revealed by reduced late endosomal vacuolation induced by the mutant pore-forming toxin ASSP, reduced intoxication by the anthrax lethal toxin and inhibition of infection by the Vesicular Stomatitis Virus.

**Conclusions/Significance:**

These results suggest that back fusion of intralumenal vesicles with the limiting membrane of late endosomes is dramatically perturbed upon cholesterol accumulation.

## Introduction

Mammalian cells harbor a complex endocytic pathway that fulfills a variety of functions such as uptake of nutrients, recycling of proteins and lipids back to the plasma membrane, down-regulation of signaling receptors, detection and destruction of pathogens [1], [2], [3]. Endosomes also extensively communicate with the biosynthetic pathway, both in an anterograde and a retrograde manner. In order to ensure proper targeting of proteins and lipids to the correct destination in the cell, sorting occurs at three stages of the pathway: the plasma membrane, the early endosomes and the late endosomes. Of these compartments, the late endosome appears to be the most complex in terms of morphology [4]. Sorting in late endosomes determines whether proteins or lipids are targeted for degradation in lysosomes, recycled to the endoplasmic reticulum or the Golgi apparatus [5], or, in specific cell types, transported to the plasma membrane [6] or secreted into the extracellular medium through the exosomal pathway [7]. Late endosomes contain tubular and cisternal regions, the later being moreover multivesicular or multilamelar. In addition, late endosomes, as early endosomes, are thought to be composed of a mosaic of domains [1], [8] that are determined by protein-protein, protein-lipid and lipid-lipid interactions. In particular, platforms organized by the small GTPases Rab7 and Rab9 have been observed [9]. In addition to this compartmentalization of the limiting membrane of the organelle–which is in contact with the cytoplasm–compartmentalization between the limiting membrane and the intralumenal membranes occurs [10]. Proteins such as the lysosomal glycoprotein Lamp1 is almost exclusively found on the limiting membrane while others such as the mannose-6-phosphate receptor are abundant in intralumenal vesicles. Lipid segregation also occurs as illustrated by the strong accumulation of the unconventionnal lipid lysobisphosphatidic acid (LBPA) on internal membranes. We have also recently shown that late endosomes contain lipid raft-like domains both on their limiting and internal membranes, but that the protein composition and physical chemical properties of these domains are different [11].

A heterogeneous class of diseases united under the name of lipid storage diseases leads to the pathological accumulation of specific lipids in late endosomes, which are subsequently thought to trigger membrane trafficking defects [12], [13]. The consequences of the accumulation of a given lipid on membrane organization, compartmentalization and function are still poorly understood. We were here interested in the consequences of late endosomal cholesterol accumulation as encountered in the Niemann Pick Type C disease (NPC) [13]. NPC is a fatal, autosomal recessive neurodegenerative disease due to mutations in the NPC1 or NPC2 genes [14]. The main biochemical manifestation in NPC is elevated late endosomal accumulation of free cholesterol [15], [16], [17] followed by an increase in sphingolipids [18]. It has been proposed that endosomal accumulation of cholesterol and sphingolipids would lead to an overload of cholesterol rich raft-like membrane domains (i.e. an increase in raft to non raft membrane ratio) and cause in a general jam in traffic through the compartment [19].

Using morphological, chemical and functional approaches, we have here analyzed the consequences of cholesterol accumulation using either NPC1 cells or negatively charged amine 3beta-(2-diethylaminoethoxy)-androstenone HCl (U18666A) [20], a drug which is well established to phenocopy mutations in the NPC1 gene. The exact targets of U18666A have not been identified and thus the mechanisms by which the drug triggers late endosome/lysosomes cholesterol accumulation remain obscure. We found that cholesterol accumulation did not lead to raft overload as anticipated. However stabilization of raft-like domains and decreased trafficking between the intralumenal vesicles and the limiting membrane of the compartment was observed associated with a drastic enlargement of compartment.

## Results

### Isolation of cholesterol-loaded late endosomes

**Figure 1:**
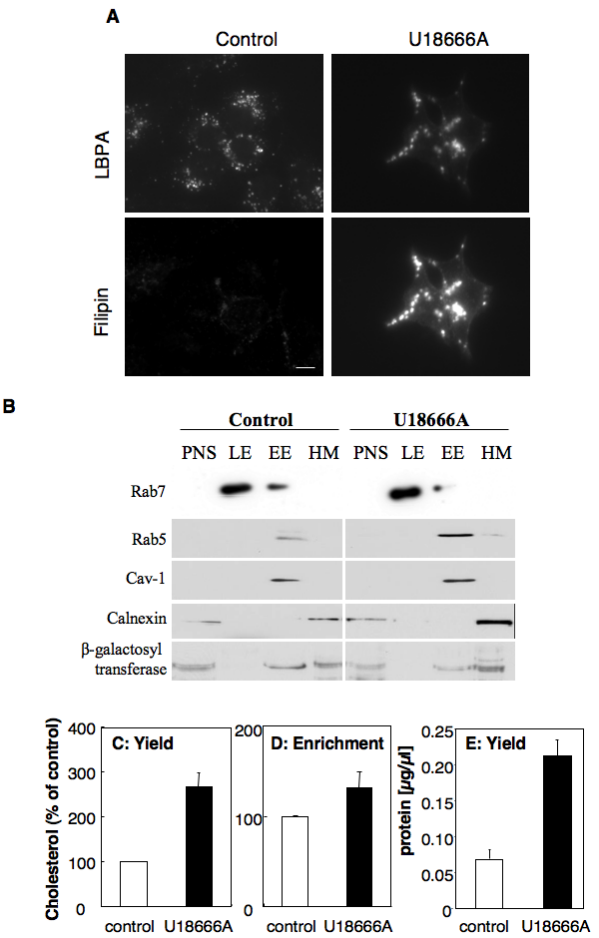
Purification of late endosomes from control and U18666A treated-BHK cells. BHK cells were treated or not with U18666A at 3 µg/ml for 18 hours. A: Cells were labeled with filipin (to detect cholesterol) and anti-LBPA antibodies. B: Cells were submitted to subcellular fractionation, as described in experimental procedures, to separate late from early endosomes. 10 µg of protein from each fraction were separated in 12.5% SDS-PAGE and blotted for the presence of the small GTPases rab7 (late endosomal marker) and rab5 (early endosomal marker), calnexin (endoplasmic reticulum marker), caveolin-1 (caveolar marker) and ß-galactosyl transferase (Golgi enzyme). An equivalent proportion (C) or an equal amount of protein (D) of the late endosomal fraction from BHK cells treated or not with U18666A (3 µg/ml for 18 hours) was submitted to lipid extraction and 1D TLC for cholesterol determination. The cholesterol was quantified by densitometry. Error bars represent the standard deviation (n = 3). E: The protein content of equivalent volume of the late endosomal fraction from BHK cells treated or not with U18666A was determined. Error bars represent the standard deviation (n = 3).

To study the effect of late endosomal cholesterol accumulation at the organellar level, we used the baby hamster kidney (BHK) cell line for which a well-established subcellular fractionation protocol is available [21], [22]. The NPC phenotype was induced using the drug U18666A [20], [23], [24]. As expected, treatment with 3 µg/ml U18666A for 18 hours led to the accumulation of cholesterol, detected using the fluorescent probe filipin, in late endosomes co-localizing with the unconventional lipid LBPA [25] (Fig 1A).

Late endosomes from control and U18666A-treated cells were then isolated. Fractionation properties were not significantly altered by cholesterol accumulation, since the small GTPase Rab7, a late endosomal marker [26], was recovered at the same low density position on the gradient as in the untreated controls (8%–25% sucrose interface) (Fig 1B). Also, U18666A did not lead to an increased contamination by other membranes since, as for control cells, the small GTPase rab5 (early endosomal marker), calnexin (endoplasmic reticulum marker), caveolin-1 (caveolar marker) and ß-galactosyl transferase (Golgi enzyme) could not be detected in the fraction (Fig. 1B).

We then analyzed the cholesterol content of purified late endosomes from control and U18666A treated cells by comparing yields (Fig. 1C, equal volumes of the late endosomal fractions were analyzed) and enrichment (Fig 1D, equal amounts of proteins from the late endosomal fractions were analyzed). As expected, the late endosomal fraction from U18666A treated cells contained significantly (2.6 fold) more cholesterol than that of control cells (Fig. 1C) in agreement with previous studies [27]. Surprisingly however, this drug-induced increase in cholesterol was small (1.3 fold, Fig 1D), when normalized to the protein content. Indeed the late endosomal fraction obtained from U18666A-treated cells contained more protein than controls (Fig. 1E). This relatively mild increase in cholesterol levels was also observed in late endosomes from cells of NPC patients [28].

### Cholesterol accumulation in late endosomes leads to an increase in compartment size

**Figure 2:**
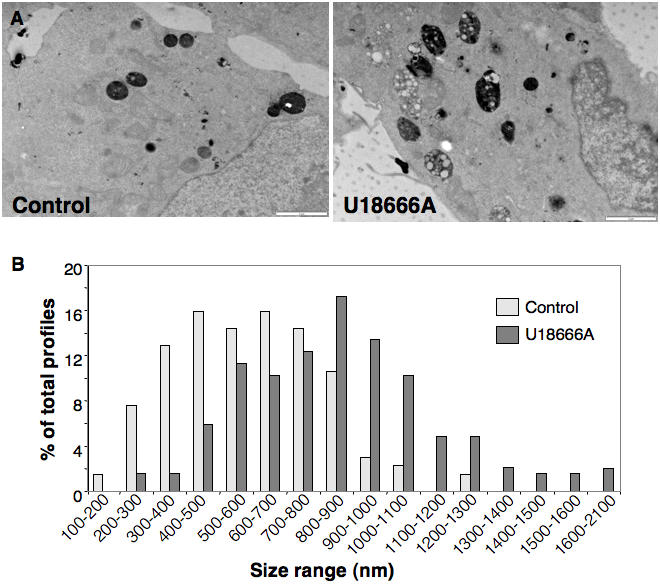
U18666A leads to an enlargement of late endosomes. A: Control and U18666A treated cells were analyzed by electron microscopy. B: The diameters of 132 late endosomes from control cells and 189 from U18666A treated cells was measured and the % of profiles for different size ranges were determined and plotted.

The fact that both the amount of cholesterol and the amount of protein increased in late endosomes of U18666A treated cells suggested that either the size or the number of late endosomes had increased. To address this issue, we compared the volume of late endosomes in control and U18666A-treated cells, after quantification by electron microscope using stereological methods. Late endosomes of control and U18666A-treated BHK cells were labeled by fluid phase uptake of horseradish peroxidase (HRP) for 60 min at 37°C followed by a 10 min chase. As shown in Fig. 2A, treatment with U18666A led to larger endocytic structures. Some late endosomes also contained large spherical lipid droplet-like structures (Fig. 2A), which are also typically found in NPC cells [24]. After stereological analysis of randomly sampled sections, the volume of late endosomes/volume of cytoplasm was estimated to be 3.05±0.72 in control cells vs. 6.82±1.18 in U18666A-treated cells, i.e. a 2.2 fold increase. Assuming no change in cell volume upon U18666A treatment, these results are remarkably similar to the increases in protein and cholesterol content of the late endosomal fraction. We also analyzed the size of HRP-labeled profiles. As shown in Fig. 2B, the distribution of sizes was significantly shifted to larger profiles upon U18666A treatment, the mean diameter of late endosomes being 589±219 nm (n = 132) for control cells and 850±290 nm (n = 186) for U18666A treated cells.

**Figure 3:**
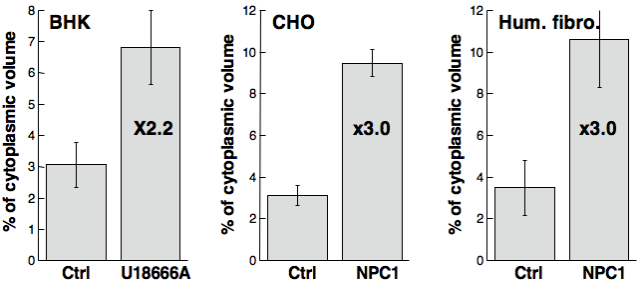
NPC1 deficiency leads to the enlargement of late endosomes. The % of cytoplasmic volume occupied by late endosomes was determined by quantitative electron microscopy on U18666A treated late endosomes, NPC1 deficient CHO cells and fibroblast from an NPC1 patient [24] in comparison to their respective controls.

These experiments thus show that U18666A-induced cholesterol accumulation in late endosomes is accompanied by a concomitant increase in the size of the compartment. We wondered whether this was also the case for cholesterol accumulation triggered by the expression of deficient NPC1 protein. To compare cells with the same genetic background, we made use of the NPC1 deficient CHO cell line CT43, and its parental line, 25RA [29]. We also compared fibroblasts from NPC1 patients to that of control human fibroblasts [24]. Both for CHO CT43 cells and NPC1 fibroblasts, the volume of late endosomes/volume of cytoplasm was increased by 3 fold when compared to their respective control (Fig. 3). Thus, whether late endosomal cholesterol accumulation was triggered by U18666A or mutations in the NPC1 protein, the size of late endosomes increased 2 to 3 fold. These findings explain the earlier observations that the intracellular accumulation of the lysosomotropic compound methylamine is increased in NPC1 patients [30].

### Effects of U18666A on the phospholipid composition of late endosomes and their DRMs

**Figure 4:**
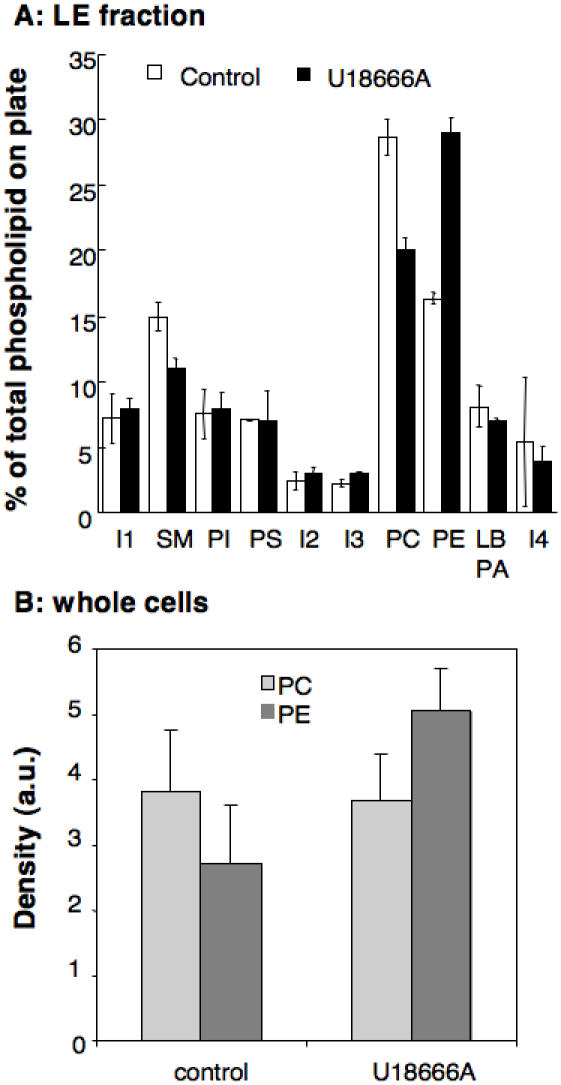
Lipid composition of late endosomes from control and U18666A treated cells. A: Late endosomes were purified from control and U18666A treated cells. An equivalent amount of protein (100 µg) from each late endosomal fraction was submitted to lipid extraction. The samples were run on two dimensional thin layer chromatography (TLC) plates for phospholipids analysis. The phospholipids were quantified by densitometry and each one expressed as percentage of the total amount of phospholipids on the plate. Error bars represent the standard deviation (n = 3). I: unidentified lipid, PI: phosphatidyl inositol, PS: phosphatidyl serine, PE: phosphatidyl ethanolamine, PC: phosphatidyl choline, LBPA: Lysobisphosphatidic acid. B: The content in total PC and PE, corresponding to an equivalent amount of protein was compared between control and U18666A treated cells.

We next analyzed the effect of U18666A on the phospholipid composition of late endosomes. Lipids were extracted and the relative proportions of phosphatidyl choline (PC), phosphatidyl ethanolamine (PE), phosphatidyl inositol (PI), phosphatidyl serine (PS), sphingomyelins (SM) and LBPA were analyzed by 2 dimensional thin layer chromatography [11], [25]. Each spot on the 2D TLC plate was quantified by densitometry and expressed as a percentage of the total intensity on the plate. The overall profile was very similar for late endosomes of control vs. drug treated cells (Fig. 4A) with the notable exceptions of a 27% decrease in sphingomyelin, in marked contrast to what has been observed in NPC1 cells [28]. U18666A treated cells also showed an inversion of the PC to PE ratio (1.75 in control vs. 0.69 in U18666A). This change in ratio reflects the global increase in cellular PE upon U18666A treatment leading also to an overall inversion of the PC/PE ratio (Fig. 4B) (PC/PE ratio 1.41 in control cells vs. 0.73 in U18666A treated cells).

**Figure 5:**
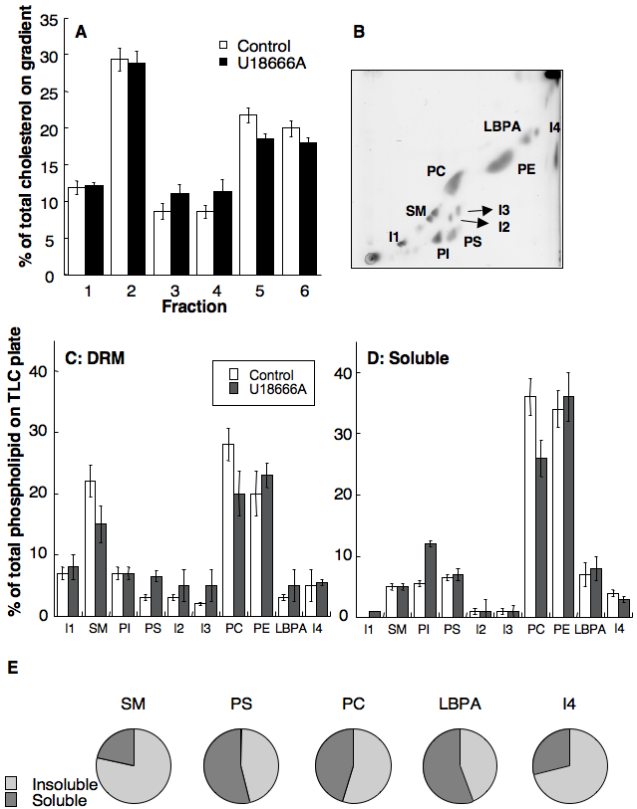
Lipid analysis of late endosomal DRMs from U18666A treated cells. DRMs were prepared from late endosomes (BHK cells +/− U18666A) by flotation on an OptiPrep gradient. Six fractions of 400 µl were collected. A: Lipids were extracted and the amount of cholesterol determined by 1D TLC. Cholesterol contents were expressed as a percentage of the total cholesterol on the gradient. Error bars represent the standard deviation (n = 3). B: After pooling fractions 1 and 2 from the top of the Optiprep gradient (corresponding to the DRMs), lipids were extracted and analyzed by 2D TLC (shown TLC corresponds to the U18666A condition). I: unidentified lipid, PI: phosphatidyl inositol, PS: phosphatidyl serine, SM: sphingomyelins, PE: phosphatidyl ethanolamine, PC: phosphatidyl choline, LBPA: Lysobisphosphatidic acid. C–D: The amount of each phospholipid in fractions 1+2 (C) and fraction 6 (D) were quantified by densitometry. Each phospholipid is expressed as percentage of the total amount of phospholipids on the TLC plate. Error bars represent the standard deviation (n = 3). E: In order to evaluate the distribution of SM, PS, PC, LBPA and I4 through out the Optiprep gradient upon fractionation of late endosomes form U18666A treated cells, the content of these lipids in fractions 1+2 and in fraction 5+6 was determined. For each lipid, the distribution between these two pools was plotted.

We next investigated whether U18666A would affect the composition/abundance of DRMs in late endosomes. The cholesterol profile along the Optiprep gradient was remarkably similar for late endosomes of U18666A treated and control cells, the low buoyant fractions 1 and 2 containing some 40% of the total cholesterol (Fig. 5A). We next analyzed the lipid composition of the DRMs fractions (fractions 1+2, Fig. 5C) in comparison to that of the detergent soluble fraction 6 (Fig. 5D). As previously observed for DRM fractions from late endosomes of control cells [11], the three major phospholipids in DRMs from late endosomes of U18666A treated cells were SM, PC and PE. These DRMs also contained the 4 unknown lipids (termed I1 to I4) detected in the DRMs of late endosomes from control cells [11]. Also the lipid composition of the soluble fraction of late endosomes from U18666A treated cells were remarkably similar to that observed for control cells (Fig. 5D). The only notable exception was a slight decrease in SM content (16% vs. 22%) in the DRM fractions and an inversion of the PE/PC ratio as also observed in Fig. 4 for the entire compartment.

### Effect of U18666A on the protein composition of late endosomal domains

**Figure 6:**
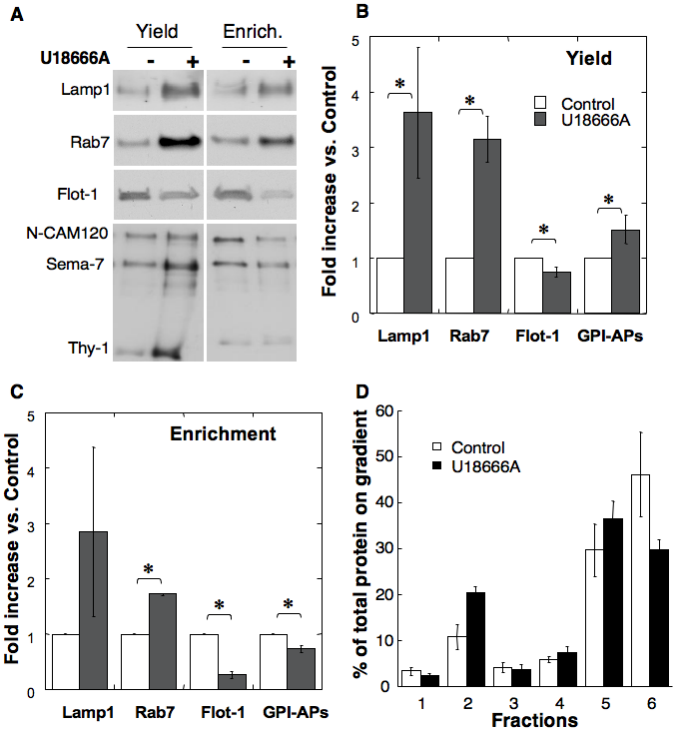
Effect of U18666A on late endosomal proteins. A: Late endosomes were isolated from control and U18666A treated BHK cells. Fractions were analyzed by SDS-PAGE either in terms of equal volume (Yield, same fraction of total late endosomes) or equal amount of protein (enrichment) and immunoblotted for the presence of Lamp-1, Rab7, flotillin-1 (Flot-1) or GPI-anchored proteins, which were revealed using an aerolysin overlay. BC: For each of these proteins, the change in abundance upon U18666A treatment vs. control was determined through quantitation by densitometry. Quantifications were performed both for analysis by Yield and by Enrichment. Error bars represent the standard deviation (n = 3). * represent statistically significant differences (p<0.05). D: DRMs were prepared from late endosomes (BHK cells +/− U18666A) by flotation on an OptiPrep gradient. Six 400 µl fractions were collected and their protein content determined. Each fraction protein content is expressed as percentage of the total amount of proteins along the gradient. Error bars represent the standard deviation (n = 3).

We next investigated the effect of U18666A on late endosomal proteins. The total amounts of Lamp-1, Rab 7 and GPI-anchored proteins (N-CAM120, semaphorin-7 and Thy-1) were increased upon U18666A treatment, with the exception of flotillin, which was slightly decreased (Fig. 6A lanes Yield: the same proportion of each late endosomal fraction was analyzed, and Fig. 6B). Therefore, with the exception of flotillin-1, the behavior of these proteins paralleled that of the total late endosomal protein population depicted in Fig. 1E. When equal amounts of LE endosomal proteins were compared (Lanes labeled Enrichment, 10 µg of protein per lane), the relative amounts of lamp1 (although not statistically significant) and Rab7 were increased upon U18666A treated (Fig. 6A and 6C). Amounts of both flotillin-1 and GPI-anchored proteins, however, were diminished (Fig. 6A and 6C).

**Figure 7:**
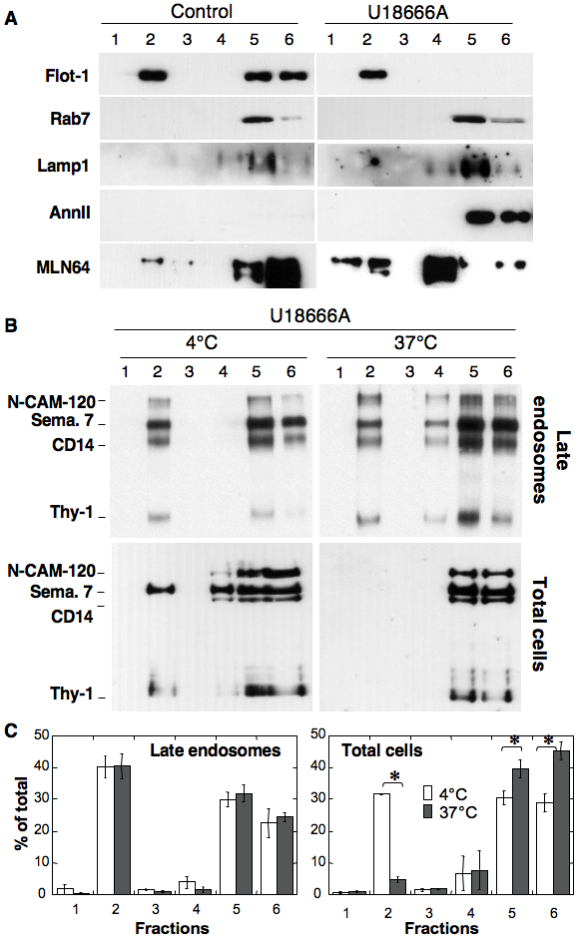
U18666A alters the detergent solubility of GPI-anchored proteins in late endosomes. A: DRMs were prepared from late endosomes (BHK cells +/− U18666A) by flotation on an OptiPrep gradient. Six fractions were collected from the top and analyzed by immunoblotting for the presence of flotillin-1 (Flot-1), Rab7, Lamp-1, annexin II (AnnII) and MLN64. B: BHK cells treated or not with U18666A. Total cells or purified late endosomes were solubilized in 1% Triton ×100 at 4°C (as in A and all other figures) or 37°C. The lysats were submitted to flotation on Optiprep gradients, 6 fractions collected and analyzed for the distribution of GPI-anchored proteins by aerolysin overlay. C: For the 4 conditions illustrated in B and for 3 independent experiments, the total GPI content of each fraction was quantified by densitometry and normalized to the total GPI-content of the gradient. Error bars represent the standard deviation (n = 3). * represent statistically significant differences (p<0.05).

When analyzing the overall protein profile on Optiprep gradients after detergent solubilization of late endosomes, a decrease in solubilized proteins was observed upon U18666A treatment with a concomitant≈2-fold increase in DRM-associated proteins (Fig. 6D). This was apparently not due to an abnormal raft association of previously non-raft proteins since both rab7 and lamp1 remained detergent soluble (Fig. 7A). A relative increase in the DRM association of flotillin-1–despite the late endosomal flotillin-1 levels (Fig. 6C)–and MLN64 was observed (fig. 7A), whereas the behavior of GPI-anchored proteins was not significantly altered (fig. 7B compared to [11]). We also analyzed the distribution of annexin II since this protein, which can associate with membranes in a cholesterol dependent manner [31], was shown to redistribute from early to late endosomes upon U18666A treatment as well as in NPC1 cells [32]. As predicted, annexin II was not detectable in control late endosomes, but was abundant and fully detergent-soluble in late endosomes from U18666A-treated cells (Fig. 7A). These observations strongly indicate that the increase in protein in late endosomal DRMs observed upon U18666A treatment is due to a specific subset of proteins.

We next wondered whether, in addition to being able to recruit more proteins, late endosomal DRMs from U18666A-treated cells had altered properties such as being more resistant to detergent extraction at 37°C instead of 4°C. Whereas this treatment led to solubilization of GPI-anchored proteins in control cells [11], the proportion of DRM associated GPI-anchored proteins was similar at 4 and 37°C for late endosomes from U18666A treated cells (Fig. 7BC). This effect of U18666A appeared to be specific to late endosomal DRMs since GPI-anchored proteins from total cell extracts (presumably reflecting the presence of plasma membrane where the bulk of GPI-anchored proteins reside) of U18666A treated cells were solubilized by Triton X-100 at 37°C (Fig 7BC).

### U18666A affects intra-endosomal trafficking

The increased resistance to detergent solubilization of late endosomal GPI-anchored proteins upon U18666A treatment raised the possibility that the dynamics of GPI-domains in intralumenal vesicles were altered—indeed, we have previously shown that GPI-anchored proteins reside mostly in intralumenal membranes [11]. To test this possibility, we analyzed the effects of U18666A on three events that rely on the dynamics of intralumenal late endosomal membranes. We studied the effects of the drug on two bacterial toxins that rely on lipid rafts for intoxication and interact with late endosomes at crucial steps of their mode of action–the mutant bacterial pore-forming toxin ASSP [22] and the anthrax toxin [33], [34]–, as well as one virus, the Vesicular Stomatitis Virus (VSV) that relies on late endosomal intralumenal vesicles for delivery of the nucleocapsid into the cytoplasm [35].

**Figure 8:**
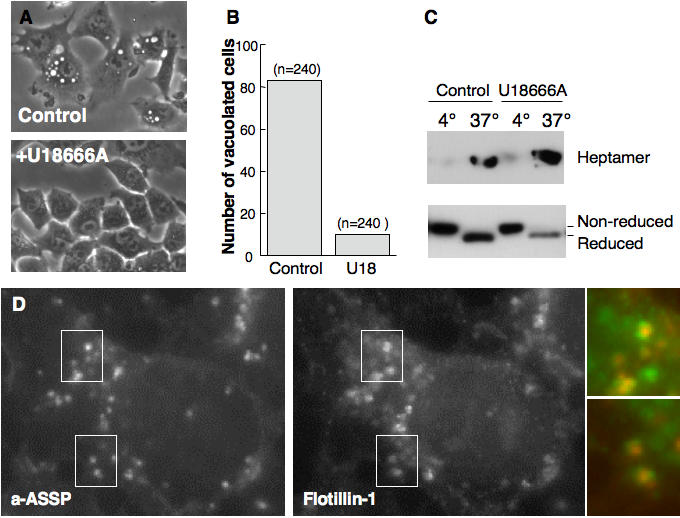
U18666A treatment protects cells against the action of ASSP. AB: BHK cells were treated with or not with U18666A for 8 hrs, followed by 9 hrs treatment, still in the presence of the drug, with the mutant aerolysin toxin ASSP (500 ng/ml). B. For each conditions, the number of vacuolated cells, out of a total of n = 240, was counted. C: BHK cells were treated with trypsin nicked ASSP (500 ng/ml) either for 1 h at 4°C or for 24 h at 37°C. For cells treated at 4°C, 20 µg of total cell extracts were analyzed for the presence of monomeric and heptameric aerolysin by non-reducing SDS-PAGE and Western blotting. Cells treated at 37°C, were submitted to subcellular fractionation to isolate late endosomes, which were analyzed for the presence of monomeric and heptameric aerolysin by non-reducing SDS-PAGE and western blotting. Monomeric ASSP underwent differential migration when reduced in late endosomes vs. non-reduced at the cell surface as previously observed [22]. D: BHK cells were treated with U18666A for 8 hrs. Alexa-ASSP was subsequently added and allowed to be internalized for 6 hrs. Cells were fixed and processed for fluorescence microscopy to detect flotillin-1 in late endosomes.

ASSP is a mutant of aerolysin that retains its capacity to bind specifically to GPI-anchored proteins but requires GPI-dependent transport to late endosomes to form channels. It indeed contains an engineered disulfide bridge that becomes reduced in late endosomes, allow the toxin to heptamerize and form channels. Channel formation, in the intralumenal vesicles as inferred by the localization of GPI-anchored proteins [11], leads to vacuolation of late endosomes [22] presumably by triggering back fusion of intralumenal vesicles with the limiting membrane. Whereas ASSP led to the appearance of cytoplasmic vacuoles in control cells, U18666A treated cells retained their normal, unvacuolated appearance (Fig. 8A and B). The lack of observed vacuolation was not due to impaired transport from early to late endosomes in U18666A treated cells since the toxin was equally reduced and heptamerized in control and U18666A treated cells (Fig. 8C). ASSP was still delivered to the intralumenal vesicles of late endosomes as suggested by the fact that internalized Alexa-labeled ASSP could be detected in punctate structures sometimes surrounded by a ring of flotillin-1 (Fig. 8D).

**Figure 9:**
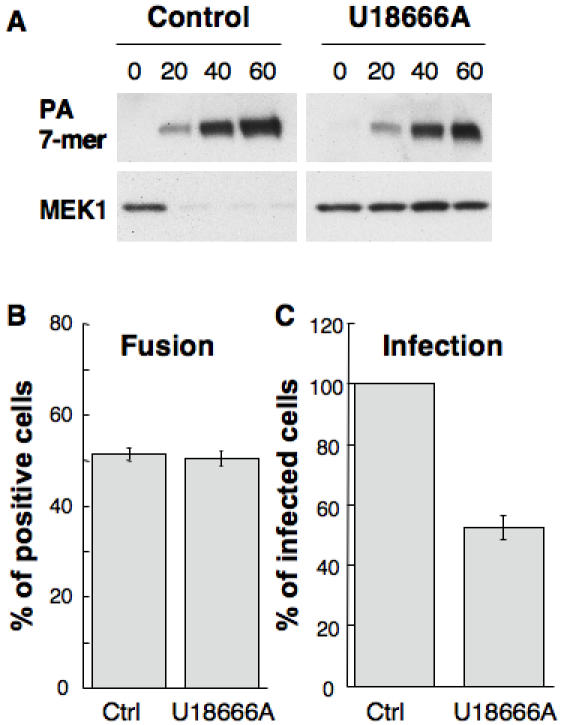
U18666A treatment protects cells against the action of anthrax toxin and from infection by the Vesicular Stomatitis Virus. A: BHK cells treated or not with 3 µg/ml U18666A were incubated 1 h at 4°C with 500 ng/ml anthrax protective antigen (PA) and 100 ng/ml lethal factor (LF) [34]. The cells were then transferred to 37°C for different periods of time in a toxin-free medium. 40 µg protein of the PNS was analyzed by western blotting against PA to detect the heptameric channel form and against MEK1, using an anti N-terminal antibody. B: BHK cells were pretreated or not during 18 hrs with U18666A and then incubated with Dil labeled VSV (0.3 MOI) at 4°C for 10 min. The temperature was then increased to 37°C. The number of cells containing fused viruses was counted 45 min post-infection. C:BHK cells were pretreated or not during 9 hrs with U18666A and then infected with VSV at an MOI of 1. After 3 hrs of VSV infect, cells were analyzed by immunofluorescence microscopy using antibodies against VSV-G. Typically, ≈70% of the cells were infected under control conditions and the number of infected cells was expressed as a percentage of the untreated control.

The anthrax lethal toxin [36] also enters cells via a raft dependent route [33] and remains associated with DRMs throughout the endocytic pathway (Abrami and van der Goot, unpublished observations). At the level of early endosomes, the channel forming subunit of the toxin, the protective antigen (PA), forms a channel in intralumenal vesicles and translocates, into the lumen of these vesicles, its enzymatic subunit, called lethal factor (LF) [34]. In order to get access to the cytoplasm, LF must then be transported to late endosomes and await back fusion of the intralumenal vesicles with the limiting membrane. To probe for alteration in DRM containing intralumenal vesicle dynamics, we investigated whether U18666A treatment would affect the release of LF into the cytoplasm by monitoring the processing of one of its targets, the MAP kinase kinase MEK1. As shown in Fig. 9A, U18666A protected cells from anthrax lethal toxin induced cleavage of MEK1. This was not due to altered internalization of the toxin since the formation of the PA channel, which forms in early endosomes, was unaltered by the drug, as monitored by the appearance of an SDS-resistant heptamer (Fig. 9A).

Finally, to further investigate the consequences of late endosomal cholesterol overload, we made use of Vesicular Stomatitis Virus (VSV), which infects cells from endosomes. The acidic endosomal pH triggers fusion of the viral envelope with endosomal membranes, thereby releasing the viral nucleocapsid into the cytoplasm and allowing infection to proceed. Le Blanc et al. [35] have recently shown that viral envelope fusion occurs preferentially with the membrane of intralumenal vesicles, and not with the limiting membrane, so that viral capsids remain hidden within the lumen of these vesicles. Back fusion, as for the anthrax toxin, allows release of the nucleocapsid to the cytoplasm and infection of the cells. To monitor viral fusion events, we used an established viral fusion assay [35]. Virions were labeled with self-quenching amounts of Dil, a fluorescent long-chain dialkylcarbocyanine dye, and bound to the cell surface at 4°C. After endocytosis at 37°C, the fusion of individual virions was revealed in the light microscope by the appearance of fluorescent spots in endosomes, due to Dil de-quenching. Treatment with U18666A had no effect on viral fusion, demonstrating that the drug did not affect transport of viral particles to acidic endosomes, or the low pH-mediated viral fusion itself. By contrast, VSV infection was significantly reduced, presumably because capsids that were released upon fusion then remained trapped within late endosomes.

## Discussion

Late endosomes are arguably the most complex station of the endocytic pathway due to their ability to communicate with other organelles, i.e. inter organellar trafficking (with Golgi, lysosomes, ER), but also due to the communication between the intralumenal vesicles and the limiting membrane, i.e. intra organellar trafficking [5]. Both proteins and lipids are responsible for generating and maintaining the compartmentalization within this compartment and its proper functioning. Our interest was to investigate the effects of late endosomal cholesterol accumulation as observed in cells from NPC patients.

Cholesterol accumulation was induced in BHK cells to allow isolation of late endosomes by subcellular fractionation. Lipid analysis revealed that the composition of late endosomes was remarkably similar in control and U18666A treated cells with the exception of a decrease in the percentage of sphingomyelin and an inversion in the PC to PE ratio. Also the fraction of detergent resistant cholesterol was unaltered suggesting that there was in fact no change in the raft to non-raft ratio. The overall amount of cholesterol was however increased as expected from the literature, but it was accompanied by an almost compensating increase in the amount of other lipids and of proteins. Quantitative electron microscopy confirmed that the late endosomal compartment was enlarged in U18666A treated cells. This was due to the cholesterol accumulation and not to some side effect of the drug since a three-fold increase in cytoplasmic volume occupied by late endosomes was also observed in a CHO cell line deficient in NPC1 and in fibroblast from an NPC1 patient.

The increase in late endosomal cholesterol however clearly did not just lead to an identical, albeit larger, compartment, as most drastically illustrated by the severity of the NPC phenotype [14]. Our analysis revealed significant difference that could contribute to explain the altered properties of cholesterol laden late endosomes. Although the increase in cholesterol was accompanied by an increase in other organellar components, the compensatory effect was not complete leading to a 30% increase in the cholesterol to protein ratio in late endosomes upon U18666A treatment. Also, although the proportion of DRMs was not altered in U18666A treated cells, these became more resistant to detergent solubilization possibly due to the increase in phosphatidyl ethanolamine. This lipid, due to its small head group, has a significantly higher melting temperature than other phospholipids [37] and thus promotes the formation of liquid ordered domains. In addition, late endosomal DRMs from U18666A treated cells showed a 2-fold increase in protein content suggesting that protein dynamics within the membranes were altered.

Previous studies have established that late endosomal motility is impaired in NPC and U18666A-treated [38], [39], [40] presumably via an inhibition of the membrane-cytosol cycle of the small GTPase Rab7, hence that inter organellar trafficking is reduced. We now observe that intra-organellar traffic is also reduced, and that dynamics of internal membranes were altered, as revealed by the (1) reduced late endosomal vacuolation induced by the mutant pore-forming toxin ASSP, (2) reduced intoxication by the anthrax lethal toxin and (3) reduced infection by Vesicular Stomatitis Virus, three events that are thought to rely on back fusion of intralumenal vesicles with the limiting membrane of late endosomes [22], [34], [35]. Such inhibition of back fusion events in U18666A-treated cells and NPC cells is expected to interfere severely with the sorting and trafficking of proteins that cycle between intraluminal and limiting membranes, as was shown for the mannose-6-phosphate receptor [24]–a situation that may well contribute to the complex pathology of NPC, and presumably of other lipidosis that are accompanied by cholesterol accumulation in late endocytic organelles [15].

### Conclusion

Pathological increases in late endosomal cholesterol, such as observed in NPC patients, led to a drastic enlargement of the compartment and alteration of its properties. Unexpectedly however, the raft to non-raft membrane ratio was not altered in late endosomes of U18666A treated cells. But, the action of toxins and viruses that target late endosome intralumenal membranes was significantly reduced, supporting the notion that cholesterol accumulation inhibits intra-endosomal membrane dynamics. Such perturbations of late endosomal membrane domains, in particular raft-like membranes, provide a reasonable explanation for altered protein and lipid trafficking through the compartment in cholesterol storage diseases.

## Materials and Methods

### Cell culture and drug treatment

Monolayer of baby hamster kidney (BHK) and Chinese Hamster Ovary (CHO) [41], [42], [43]. For 3beta-(2-diethylaminoethoxy)-androstenone HCl (U18666A) treatment, the cell culture medium was removed 4 hours later after plating and fresh medium containing U18666A at 3 µg/ml was added for 18 hours.

### Subcellular fractionation

Late endosomal fraction was prepared as described [21], [22]. Briefly, BHK cells were homogenized, a post-nuclear supernatant was prepared and adjusted to 40.6% sucrose, 3 mM imidazole, pH 7.4, loaded at the bottom of an SW41 tube, and overlaid sequentially with 35 and 25% sucrose solutions in 3 mM imidazole, pH 7.4, and then homogenization buffer (HB; 8.5% sucrose, 3 mM imidazole, pH 7.4). The gradient was centrifuged for 90 min at 35 000 rpm. Early and late endosomal fractions were collected at the 35/25% and 25%/HB interfaces respectively.

### Isolation of DRMs from late endosomal fractions

DRMs were prepared from late endosomes as described by [22]. Briefly late endosomes were diluted four times, sedimented by centrifugation (TLS.55 Beckman rotor, 30 min, 55 000 r.p.m.) and resuspended in 200 µl of lysis buffer (25 mM Tris-HCl pH 7.4, 150 mM NaCl, 5 mM EDTA) in the presence of Complete, a cocktail of protease inhibitors (Roche) and 1% Triton X-100. After 20 min of incubation at 4°C, the lysat was adjusted to 40% OptiPrep (Nycodenz), overlaid with a 30% and 0% OptiPrep cushions and centrifuged for 2h centrifugation at 55 000 rpm (4°C) using a TLS.55 rotor. Six fractions were collected from the top and precipitated with 6% trichloroacetic acid in the presence of sodium deoxycholate as a carrier. When indicated, DRMs were prepared after samples solubilization in Triton X-100 by high-speed centrifugation for 30 min at 55'000 using a tabletop ultracentrifuge.

### Phospholipid and cholesterol analysis

Lipids of late endosomes and late endosomes fractions obtained with OptiPrep gradient centrifugation were extracted in CHCl3/MeOH and then separated by two-dimensional thin layer chromatography (TLC) for phospholipids analysis [25], [44]. The first dimension was run with chloroform, methanol, 32% ammonia (65∶35∶5, v/v) and the second with chloroform/acetone/methanol/acetic acid/water (50∶20∶10∶12.5∶5), v/v). Phospholipids were revealed by burning the TLC plate at 160°C after immersion in 1.5 mM cupric acetate-8% H_3_PO_4_ solution.

For cholesterol analysis, lipids were extracted as above, analyzed on a one-dimensional TLC in heptane/ethylether/acetic acid (18∶6∶2, v/v) and stained with copper. Both cholesterol and phospholipids were quantified by densitometry using the ScanAnalysis software.

### Immunoblotting, aerolysin overlays and protein quantification

Proteins were separated by SDS-PAGE using 12.5% acrylamide gels unless stated otherwise and transferred onto a nitrocellulose membrane. Western blots were revealed with SuperSignal Chemiluminescence (Pierce) and quantified by densitometry. Aerolysin overlays were performed as described by [45]. Protein contents of cellular fractions were determined using bicinchroninic acid (BCA, Pierce).

### Vesicular Stomatitis virus (VSV) infection and toxin treatments

Before infection BHK cells grown on glass cover slips were treated during 9 hours with U18666A. Cells were then incubated 1 h at 4°C with VSV (1 MOI) in MEM pH 7.4 followed by 3 h at 37°C, in the presence of the drug. Finally cells were fixed and analyzed by immunofluorescence and infection was quantified by counting the number of cells expressing either newly synthesized VSV-G protein or GFP [35]. Treatment of BHK cells with the mutant pore-forming toxin ASSP and anthrax lethal toxin were performed as described by [22], [34] respectively.

### Fluorescence Microscopy

Cells grown on cover slips were fixed with 3% paraformaldehyde for 20 min at room temperature and saturated with 10% phosphate-buffered saline-fetal calf serum (PBS-FCS) for 20 min. Cholesterol was labeled with 50 µg/ml Filipin in 10% PBS-FCS. For flotillin-1 labeling cell PFA fixed cells were permeabilized with saponin and stained with a polyclonal antiflotillin-1 antibody [22]. Cells were mounted on microscopy slides. Images were acquired using the IPLab Spectrum software (Scientific Image Processing).

### Electron microscopy

BHK cells were treated with 3 µg/ml U18666A for 18 hours or were incubated under identical conditions in the absence of the drug. The cells were then incubated with HRP (10 mg/ml) for 60 min at 37°C followed by a further 10 min incubation without HRP to allow the HRP to chase out of early endosomal compartments. The cells were then fixed with 2.5% glutaraldehyde in PBS and processed for peroxidase detection and EM embedding using standard conditions. Digital images of thin sections were captured at random while scanning across the grid in a regular unbiased manner. Images were overlaid with a lattice grid generated in the imaging program (SIS SoftImaging Munster, Germany). Volume of the HRP-containing compartments was quantitated by point counting using double lattice grids to relate points over HRP-labeled profiles to the cytoplasmic volume using standard methods [46].
